# Pubertal timing, body dissatisfaction and self-image: a prospective cohort study

**DOI:** 10.1136/bmjopen-2024-092212

**Published:** 2025-08-04

**Authors:** Dana Tarif, Jon Heron, Abigail Fraser, Carol Joinson

**Affiliations:** 1 Department of Population Health Sciences, University of Bristol, Bristol, UK

**Keywords:** Adolescent, MENTAL HEALTH, Body Mass Index

## Abstract

**Abstract:**

**Objective:**

Early pubertal timing has been linked to heightened body dissatisfaction, but previous studies have focused on girls, with small sample sizes and lacking objective measures of pubertal timing. The objective of this study was to examine the association between pubertal timing (age at peak height velocity [aPHV] and age at menarche [AAM] for girls) and body dissatisfaction and self-image in mid-adolescence (age 14).

**Design and setting:**

Prospective cohort study in the UK.

**Participants:**

6644 participants (41% male) from the Avon Longitudinal Study of Parents and Children.

**Outcome measures:**

Outcomes were measured using the Satisfaction and Dissatisfaction with Body Parts Scale and Self-Image Profile at age 14. Multivariable regression models were adjusted for socioeconomic status and prepubertal body mass index (BMI).

**Results:**

In boys, later aPHV was associated with higher body dissatisfaction (b=0.13 (95% CI 0.09 to 0.18)). In girls, later aPHV was associated with lower body dissatisfaction, but this was attenuated after adjusting for BMI (b=−0.03 (95% CI −0.07 to 0.01)). A negative association was found between AAM and body dissatisfaction (b=−0.06 (95% CI −0.09 to –0.02)). Later aPHV in girls was associated with increased odds of feeling good-looking (OR=1.09 (95% CI 1.01 to 1.19)) and lower odds of feeling different from others (OR=0.91 (95% CI 0.83 to 1.00)). No associations between aPHV and self-image were found in boys.

**Conclusions:**

These findings highlight the need for targeted interventions for adolescent body dissatisfaction.

**Strengths and limitations of this study**

- Used a large prospective cohort study with robust data collection methods.
- Assessment of an objective measure of pubertal timing (age at peak height velocity) in both sexes.
- Generalisability may be limited due to under-representation of ethnic minorities in the cohort, impacting cross-cultural applicability.
- Potential for selection bias due to attrition and exclusion of participants with missing data, despite efforts to address this through multiple imputation.

## Introduction

 Body dissatisfaction, defined as negative thoughts and feelings towards one’s own body,^1^ is prevalent in adolescence and appears to be more common among girls than boys.^2^ Body dissatisfaction is associated with an increased risk of adverse outcomes, including disordered eating behaviours and depression.^3^ Most research on body dissatisfaction has focused on girls, while research on boys is sparse.^4^

Puberty is a sensitive period for the development of body image.^5^ Girls, on average, experience onset of puberty earlier than boys, with the mean age of girls at 11 years and boys at 13 years.^6^ However, there is interindividual variability in the timing of puberty, both between and within sexes. The early timing hypothesis (or developmental readiness hypothesis) posits that early maturing individuals may experience a rapid physical development prior to cognitive and emotional development, placing them at risk for adverse psychological and behavioural outcomes.^7^ The off-timing hypothesis (or developmental deviance hypothesis) proposes that any deviation from the normative timing of puberty can result in psychological distress and adverse outcomes.^8^ A growing body of research has identified robust associations between early pubertal timing and adverse outcomes in both boys and girls, including for depressive symptoms and disordered eating.^9^

Several studies have examined the association between pubertal timing and body dissatisfaction in girls.^9 10^ Early pubertal timing in girls has been found to be associated with greater body dissatisfaction in a number of cross-sectional studies^11^,^13^ and some longitudinal studies.^14^,^16^ However, one longitudinal study found no evidence of an association between pubertal timing, measured by the pubertal development scale and body dissatisfaction in girls.^17^ Only a few of these studies controlled for important confounders, such as body mass index (BMI).^11 13 14^ Research on boys is more limited. Some cross-sectional studies^12 16^ and one longitudinal study^17^ have found that later pubertal timing in boys is associated with greater body dissatisfaction. Conversely, another longitudinal study found early pubertal timing was associated with increased appearance anxiety in boys,^15^ while other studies reported no association in boys.^18 19^

One of the main limitations of previous research is the absence of an objective measure of pubertal timing across both sexes. In girls, age at menarche (AAM) is often used as an objective and salient indicator of puberty onset, while studies in boys often use self-reported perceived timing of puberty.^12 20^ This introduces potential biases as self-reported perceptions are affected by social comparison and subjectivity. Previous studies are also limited by small sample sizes^9^ and incomplete confounder adjustment, particularly lack of adjustment for prepubertal BMI.^17^ Age at peak height velocity (aPHV), defined as the age at which height is increasing at the fastest rate (the adolescent ‘growth spurt’), is an objective indicator of pubertal timing applicable to both sexes.^21^ aPHV provides a non-invasive method of measuring pubertal timing and has been shown to correlate strongly with other measures of pubertal timing, including AAM.^21^

The primary aim of the present study is to examine the association of pubertal timing (aPHV, AAM) with body dissatisfaction and self-image in mid-adolescence in both sexes using data from a large, prospective cohort. Self-image is intricately linked with body dissatisfaction, and investigating the relevant components of self-image provides a more comprehensive understanding of any association with pubertal timing. The study focused on body dissatisfaction and self-image in mid-adolescence (at age 14) because this period coincides with a high level of interindividual variation in pubertal maturation.

## Method

### Participants

**Figure 1:**
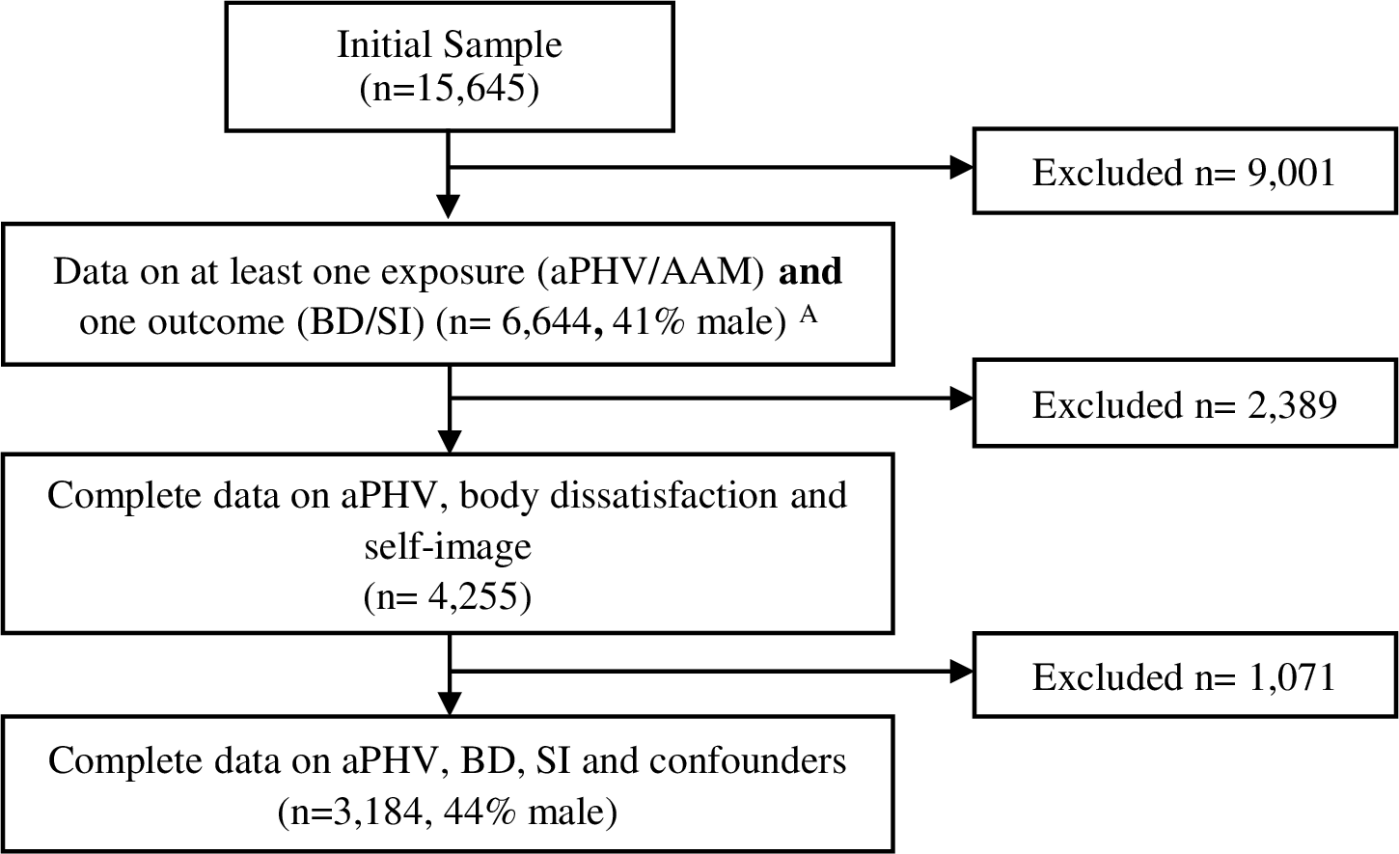
Participant flow chart. ^A^Complete data for each variable: aPHV (n=8412), AAM (n=4303), body dissatisfaction (n=4303) and self-image (n=6866). AAM, age at menarche; aPHV, age at peak height velocity; BD, body dissatisfaction; SI, self-image.

The Avon Longitudinal Study of Parents and Children (ALSPAC) is a birth cohort that originally recruited pregnant women (n*=*14 541) residing in Avon, UK with expected dates of delivery 1 April 1991 to 31 December 1992. Of the initial pregnancies, there were 14 676 fetuses, 14 062 were live births and 13 988 children who were alive at 1 year of age. When the oldest children were approximately 7 years of age, an attempt was made to increase the original sample by recruiting eligible individuals who did not join the study. This resulted in a total sample size of 15 454 pregnancies (15 658 fetuses, 14 901 alive at 1 year of age) when using data after the age of seven. Due to confidentiality reasons, data on 13 triplets/quads are not provided, resulting in 15 645 cases.^22^,^24^ Both mothers and children have been followed up with regular questionnaires and research clinics. Participants with valid aPHV data were eligible for inclusion in the study (n=8412, see figure 1).

### Exposures: pubertal timing

aPHV and AAM were used as indicators of pubertal timing. aPHV was derived from height measurements taken from clinic assessments at various timepoints between ages 5 and 20 using Superimposition by Translation and Rotation analysis, described in the detail elsewhere^25^ (online supplemental material S1). AAM (in years and months) was obtained from clinic reports and postal questionnaires administered at various timepoints, as described elsewhere.^26^

### Outcomes: body dissatisfaction and self-image at age 14

Participants provided data on body dissatisfaction and self-image in two separate questionnaires completed at approximately 14 years old (M_age_±SD=14.1 ± 0.2 and 14.0±0.1, respectively). Body dissatisfaction was assessed using an adapted version of the Satisfaction and Dissatisfaction with Body Parts Scale^27^ (available in online supplemental material S2). The original scale asked respondents about 24 aspects of the body, which were condensed to 11 aspects in the adapted version due to the repetition of similar body parts. Participants were asked to indicate their level of satisfaction with 11 body parts (weight, figure, body build or breasts, stomach, waist, thighs, buttocks, hips, legs, face and hair) using a 5-point scale from ‘extremely satisfied’ (1) to ‘extremely dissatisfied’ (5). Questions differed slightly in the boys’ and girls’ versions of the questionnaire (‘body build’ in the boys’ questionnaire was replaced with ‘breasts’ in the girls’ version). In line with previous research,^28^ a continuous score was derived ranging from 11 to 55, with higher scores indicating greater dissatisfaction. The adapted version of the scale has shown high reliability in both males (α=0.95) and females (α=0.90).^29^ A summary of responses to each individual item and internal reliability analysis is provided in the online supplemental material S5.

The self-image profile^30^ assessed how respondents identify themselves against 25 attributes relating to self-image, such as ‘kind’, ‘funny’ and ‘hard working’. We selected three of these items due to their relationship with body image, puberty and developmental deviance: how often the respondent felt (a) confident, (b) good-looking and (c) different from others, rated from ‘always’ (1) to ‘never’ (5). Responses were dichotomised to create binary scores for the analysis, due to the low responses on the extreme ends of the Likert scale.^31^

### Confounders

The analysis was adjusted for potential confounders which were identified based on empirical evidence about factors that are associated with both timing of puberty and body dissatisfaction. They included socioeconomic status (SES), measured by maternal education (certificate of secondary education/vocational qualifications/none; O-levels; A-levels and above) and occupational social class (manual vs non-manual), measured during the antenatal period, and home ownership status (renter vs owned/privately rented) and major financial problems (experienced in first 5 years of child’s life vs none), measured at various timepoints from birth to 10 years old. Detailed descriptions of the coding of each item are available in the online supplemental material S1. BMI at age 9 was calculated based on height and weight measurements obtained from clinics and questionnaire data when clinic data were missing.

### Statistical analysis

Analyses were stratified by sex due to the disparities in the timing of puberty and prevalence of body dissatisfaction between boys and girls.^17^ The potential for non-linear relationships was explored using quadratics and polynomial regression, and there was no evidence of departure from linearity (available on request). Associations between aPHV and body image at age 14 were examined using linear regression analysis, adjusting for confounders. Analyses were also carried out to examine the association between females’ AAM and body dissatisfaction, using linear regression. In order to make comparisons between aPHV and AAM, both exposures were transformed into standardised scores (z scores). Body dissatisfaction was also transformed into standardised scores (z scores), therefore, the units for the effect estimates were SDs. Logistic regression was used to examine the associations between aPHV and the three items from the self-image profile. Supplementary analyses were carried out to examine the association between aPHV and each individual item of the body dissatisfactions scale, using logistic regression (online supplemental materials S6 and S7). All analyses were carried out using Stata V.17.

### Missing data

There were 6644 participants with data on at least one of the exposure variables (aPHV/AAM) and at least one of the outcome variables (body dissatisfaction/self-image). Of these, only 3184 had complete data on aPHV, both outcomes and all confounders. Missing data were imputed using multiple imputation by chained equations.^32^ 100 imputed datasets were generated, and analyses were pooled according to Rubin’s rules.^33^ Relevant auxiliary variables were included in the imputation model (online supplemental material S1), alongside all the variables used within the analyses. Imputations were carried out separately for males and females and a separate imputation was carried out for the dataset looking only at AAM. Imputed results are presented as the main results and results from the complete case analysis are available in the online supplemental materials S3 and S4.

### Patient and public involvement

Patients and/or the public were not involved in the design, conduct, reporting or dissemination plans of this research.

## Results

Mean aPHV in the imputed dataset was 13.4 years (SD 0.9) in males and 12.0 years (SD 0.8) in females. The proportions or mean values for various characteristics compare reasonably across the different samples, shown in table 1. Table 2 shows the distribution of all variables included in the analysis, by sex. The mean body dissatisfaction score was higher among girls, who were also more likely to report lower self-image, including not feeling confident or good-looking and feeling different from others compared with boys.

**Table 1:** Characteristics across different samples (percentage/mean±SD)

	Whole sample up to N=15 645	Complete case N=3184	Imputed sample–aPHV up to N=6644	Imputed sample–AAM (females only) up to N=3889
Male sex	51.1%	43.8%	41.5%	–
Renter/non-home owner	15.6%	6.03%	9.4%	10.8%
Maternal education (Ref: >O levels)				
<O levels	21.9%	14.4%	17.3%	18.5%
O levels	40.5%	39.1 %	39.4%	39.2%
Major financial problems: yes	26.4%	23.7%	25.2%	26.6%
Manual social class	19.4%	10.1%	13.5%	14.7%
BMI at 9 (kg/m^2^)	17.7±3.00	17.6±2.76	17.7±2.89	17.9±3.00

AAM, age at menarche; aPHV, age at peak height velocity; BMI, body mass index.

**Table 2:** Distribution (proportions and mean (SEs)) of variables in imputed dataset (N=6664)

	Males	Females
Continuous variables (mean)		
aPHV (years)	13.4 (0.16)	12.0 (0.13)
AAM (years)	-	12.7 (0.19)
Body dissatisfaction*	24.3 (0.17)	28.8 (0.16)
BMI at 9	17.4 (0.05)	17.9 (0.05)
Binary variables (%)		
Self-image profile		
Not confident	7.7%	11.5%
Not good looking	16.9%	26.7%
Different from others	16.0%	20.0%
Renter/non-home owner (ref: home owner)	7.9%	11.7%
Maternal education (Ref: <O Levels)		
O levels	40.0%	39.2%
>O levels	43.6%	41.1%
Major financial problems: yes	23.4%	26.7%
Social class: manual (Ref: non-manual)	12.0%	15.6%

* Higher score indicates greater dissatisfaction.

AAM, age at menarche; aPHV, age at peak height velocity; BMI, body mass index.

### Body dissatisfaction

Table 3 shows the adjusted and unadjusted results for the association between pubertal timing (aPHV and AAM) and body dissatisfaction. In males, a later aPHV was associated with greater body dissatisfaction; a 1 SD increase in aPHV was associated with a 0.13 SD increase in the body dissatisfaction score (95% CI 0.09 to 0.18) in the fully adjusted model. In females, there was evidence for an inverse association between aPHV and body dissatisfaction in the unadjusted model; a 1 SD increase in aPHV was associated with a 0.14 SD decrease in the body dissatisfaction score (95% CI −0.17 to –0.1). This association remained after adjusting for the SES variables but was attenuated after further adjusting for BMI in the fully adjusted model (b***=***−0.03 (95% CI −0.07 to 0.01)). In comparison, in the analysis with AAM as the measure of pubertal timing, a 1 SD increase in AAM was associated with a 0.15 SD decrease in the body dissatisfaction score (95% CI −0.15 to –0.11) in the model adjusted for SES variables. After further adjustment for BMI, the effect was weakened (b=−0.06 (95% CI −0.09 to –0.02)), but remained.

**Table 3:** Linear regression coefficients for the association between pubertal timing (aPHV and AAM) and body dissatisfaction at age 14

	Unadjusted		Adjusted for SES*		Adjusted for SES* and BMI	
	Coefficient (95% CI)	P value	Coefficient (95% CI)	P value	Coefficient (95% CI)	P value
Exposure=aPHV (n=6644)						
Males body dissatisfaction	0.05 (0.002 to 0.09)	0.039	0.05 (0.01 to 0.09)	0.025	0.13 (0.09 to 0.18)	<0.001
Females body dissatisfaction	−0.14 (−0.17 to −0.10)	<0.001	−0.14 (−0.17 to −0.10)	<0.001	−0.03 (−0.07 to 0.01)	0.105
Exposure=AAM (n=3889)						
Body dissatisfaction	−0.15 (−0.18 to −0.12)	<0.001	−0.15 (−0.18 to −0.11)	<0.001	−0.06 (−0.09 to −0.02)	0.001

1 SD increase in aPHV/AAM is equal to the equivalent SD change in body dissatisfaction shown in the table.

* SES variables include maternal education, social class, home ownership and financial problems.

AAM, age at menarche; aPHV, age at peak height velocity; BMI, body mass index; SES, socioeconomic status.

We explored the associations between standardised aPHV and satisfaction with individual body parts from the body dissatisfaction scale, see online supplemental materials S6 and S7. In girls, later puberty was associated with a reduced odds of dissatisfaction with legs (OR: 0.85 (95% CI 0.76 to 0.95)), buttocks (OR: 0.83 (95% CI 0.74 to 0.94)) and thighs (OR: 0.82 (95% CI 0.74 to 0.91)), while also an increased odds of dissatisfaction with breasts (OR: 1.31 (95% CI 1.16 to 1.47)) and figure (OR: 1.12 (95% CI 1.00 to 1.25)). In males, later puberty was associated with increased odds of dissatisfaction with items such as weight (OR: 1.30 (95% CI 1.12 to 1.51)), chest (OR: 1.21 (95% CI 1.05 to 1.38)), stomach (OR: 1.34 (95% CI 1.17 to 1.54)). These analyses also revealed there was no association between aPHV and items such as hair or face, in both sexes.

### Self-image

Table 4 shows the adjusted and unadjusted associations between pubertal timing and items of the self-image profile. In females, a 1 SD increase in aPHV was associated with a 9% increase in the odds of reporting feeling good-looking in the model adjusting for SES and BMI (95% CI 1.01 to 1.19). A 1 SD increase in aPHV was associated with a 13% decrease in the odds of reporting feeling different from others (95% CI 0.80 to 0.95) in the model adjusted for SES variables, but this association was attenuated in the model adjusted for BMI (OR: 0.91 (95% CI 0.83 to 1.00)). In males, we found no evidence of an association between aPHV and all three items from the self-image profile.

**Table 4:** ORs for the association between aPHV and self-image profile items at age 14 (n=6644)

	Unadjusted		Adjusted for SES*		Adjusted for SES* and BMI	
	OR (95% CI)	P value	OR (95% CI)	P value	OR (95% CI)	P value
Males						
Confident	1.01 (0.88 to 1.17)	0.869	1.01 (0.87 to 1.17)	0.891	0.98 (0.84 to 1.14)	0.791
Good looking	1.06 (0.96 to 1.18)	0.269	1.05 (0.95 to 1.16)	0.360	0.99 (0.89 to 1.11)	0.898
Different from others	0.95 (0.85 to 1.05)	0.307	0.96 (0.86 to 1.06)	0.408	0.97 (0.87 to 1.08)	0.570
Females						
Confident	1.09 (0.98 to 1.21)	0.130	1.08 (0.97 to 1.21)	0.152	1.05 (0.93 to 1.18)	0.453
Good looking	1.15 (1.06 to 1.24)	<0.001	1.15 (1.06 to 1.24)	<0.001	1.09 (1.01 to 1.19)	0.036
Different from others	0.87 (0.80 to 0.95)	0.002	0.87 (0.80 to 0.95)	0.001	0.91 (0.83 to 1.00)	0.056

1 SD increase in aPHV is equal to the equivalent OR change shown in the table.

* SES variables include maternal education, social class, home ownership and financial problems.

aPHV, age at peak height velocity; BMI, body mass index; SES, socioeconomic status.

## Discussion

### Summary of main findings

This is the first prospective study to examine the associations between pubertal timing, body dissatisfaction and self-image in adolescence within both sexes. In girls, later pubertal timing (later aPHV and later AAM) was associated with lower body dissatisfaction; however, these associations were attenuated following adjustment for prepubertal BMI. In boys, the opposite pattern emerged; later pubertal timing (later aPHV) was associated with increased body dissatisfaction, and this association was strengthened after adjusting for BMI. Examining self-image, earlier maturing girls were less likely to feel good-looking and more likely to feel different from others, including when adjusting for BMI. In boys, there was no evidence of an association between aPHV and aspects of self-image in the fully adjusted model.

### Strengths, limitations and methodological considerations

Major strengths of this study include the use of a large prospective cohort, objective measures of pubertal timing in both sexes, validated questionnaires on body dissatisfaction and self-image, and adjustment for pertinent confounders. The use of aPHV in both sexes represents a key strength, as previous research has focused primarily on females only. The interpretation of these findings must also be considered alongside the limitations. Attrition and exclusion of participants due to missing data is a common limitation of many cohort studies which may introduce the potential for selection bias, considering the socioeconomically disadvantaged profile of excluded participants. Lower SES is associated with both earlier pubertal timing^34^ and greater body dissatisfaction^35^ in both sexes. Multiple imputation was used to address potential bias due to missing data and to increase statistical power, and the analysis of the imputed data was compared with the complete case analysis. Generalisability is also limited, given the cohort’s underrepresentation of ethnic minorities, impacting cross-cultural applicability. Cross-cultural generalisations can be problematic when examining body image due to differences in perceived ideal body shapes across cultures.^17^ Additionally, it is important to acknowledge the evolution of societal norms regarding body shapes and ideals over time, which may impact the interpretation of our findings.^36^

The use of BMI as a confounder could be considered a potential limitation. While high BMI is a widely used measure of childhood overweight and obesity, it is limited in its inability to distinguish between lean mass and fat mass, which can lead to a misclassification of obesity in adolescents.^37^ When explaining the association between onset of puberty and body dissatisfaction in girls, mechanistic explanations often refer to increases in fat mass and body fat percentage, rather than lean mass. Future research should examine whether body fat percentage also attenuates this association. While adjustment for BMI had a notable impact on estimates, adjustment for SES had minimal effect. This may reflect the more direct relationship between BMI and body image concerns, whereas SES may exert a more indirect influence. Although previous research has found that lower SES is associated with earlier pubertal timing^34^ and greater body dissatisfaction,^35^ these associations are often modest and may not fully capture the psychosocial pathways relevant to body image. Residual confounding by unmeasured factors, such as early life stress and adverse childhood experiences, may also play a role.

### Comparison within existing research

The sex differences in the association of pubertal timing and body dissatisfaction observed in this study are consistent with previous research in boys and girls.^12 17^ Previous research has also reported attenuation of this association by BMI.^13 14^ Previous research using composite measures of self-esteem or self-image found that early maturing girls reported lower levels of self-esteem.^38^ Our study extends prior research by dissecting individual components of self-image, providing novel insights into the nuanced dynamics within each sex.

In girls, associations between pubertal timing and body dissatisfaction were stronger and more consistent when using AAM compared with aPHV. This may be because AAM is a more salient and socially meaningful marker of pubertal timing, indicating the onset of reproductive capacity and is often seen as a key milestone in female maturation.^39^ In contrast, aPHV captures a broader growth process which may be less obvious to the individual or peers, and therefore, less psychologically salient.^40^ As a result, aPHV may have a weaker association with body image concerns, helping explain the observed differences in effect sizes.

Supplementary analyses examining the individual components of body dissatisfaction revealed that pubertal timing appears to be related to items more typically associated with weight concerns, such as thighs, figure and buttocks, rather than face or hair. These analyses highlighted that, particularly in girls, the association between pubertal timing and dissatisfaction with different body parts was not always in the same direction. Although later pubertal timing in girls was associated with lower overall body dissatisfaction, later puberty was associated with higher dissatisfaction with breasts and figure. This may reflect pressures related to self-objectification, whereby girls evaluate their physical development against societally reinforced ideals of femininity, potentially leading to dissatisfaction.^41^ Exploring the sources of body dissatisfaction, our findings align with existing literature indicating distinct pressures on adolescent girls to be thin and on boys to be muscular.^36 42^

Adjusting for BMI attenuated associations between pubertal timing and body dissatisfaction in girls but strengthened this association in boys, suggesting BMI may play a different role in shaping body dissatisfaction between the sexes. In girls, the attenuation supports previous research showing that higher BMI is associated with both earlier pubertal timing and greater body dissatisfaction,^35 43^ indicating that BMI may partly explain this association. In contrast, in boys, adjustment for BMI strengthened the association between pubertal timing and body dissatisfaction. This suggests that BMI may have obscured the strength of the association in unadjusted models. Later maturing boys typically have a lower BMI and may perceive themselves as too lean or underdeveloped compared with peers, which can contrast with the cultural ideal of muscularity.^42 44^ This highlights the importance of considering sex-specific body ideals and the complex role of BMI in understanding adolescent body image.

The finding of negative outcomes associated with early pubertal timing in girls is most consistent with the early timing hypothesis.^7^ Early maturing girls experience changes such as increased adiposity and changes in weight distribution before their peers, which misaligns with the reported desire for a thin-ideal body image, resulting in a vulnerability to experiencing body dissatisfaction.^36^ In contrast, the finding that late maturing boys experience greater body dissatisfaction supports the off-timing hypothesis. Late maturing boys experience pubertal changes, including the adolescent growth spurt, at a later age than their peers and may display body dissatisfaction.

## Conclusions

Our findings provide evidence that early pubertal timing in girls and late pubertal timing in boys is associated with increased body dissatisfaction during adolescence. Future research is needed to examine the causal pathways underlying these associations, potentially employing methods such as Mendelian randomisation. Moreover, further research using data from more recent cohorts will highlight whether these associations have changed over time. Identifying groups at risk of exhibiting body dissatisfaction holds potential for targeted body image interventions aimed at reducing body dissatisfaction and fostering body acceptance. By identifying individuals at risk of developing negative body image perceptions early on, interventions can potentially prevent the onset of disordered eating behaviours and unhealthy weight management practices.

## Supplementary material

10.1136/bmjopen-2024-092212 online supplemental material 1

## Data Availability

Data may be obtained from a third party and are not publicly available.
