# Supplementary material for: Pubertal timing, body dissatisfaction and self-image: a prospective cohort study
Source: BMJ Open. 2025 Aug 4;15(8):e092212. doi: 10.1136/bmjopen-2024-092212 (PMC12323538; doi:10.1136/bmjopen-2024-092212)

**Supplementary Material**

[S1. Coding of Variables 2](#_Toc173232585)

[S2. Satisfaction and Dissatisfaction with Body Parts Scale, adapted by ALSAPC 7](#_Toc173232586)

[S3. Complete Case: Linear Regression Coefficients for the association between pubertal timing (aPHV or AAM) and body dissatisfaction. 8](#_Toc173232587)

[S4. Complete Case: Logistic Regression Odds Ratios for the association between aPHV and self image profile questions in males and females (N= 3,184). 8](#_Toc173232588)

[S5. Summary of individual items of the body dissatisfaction scale and Internal Reliability Analysis 9](#_Toc173232589)

[S6. Association between standardised aPHV and individual items of body dissatisfaction scale in Males in complete sample (N=3184). 10](#_Toc173232590)

[S7. Association between standardised aPHV and individual items of body dissatisfaction scale in Females in complete sample (N=3184). 11](#_Toc173232591)

# S1. Coding of Variables

| Variables | ALSPAC  Var Name | Question | Response | Final Coding |
| --- | --- | --- | --- | --- |
| Exposure Variables | | | | |
| Age at Peak Height Velocity (aPHV) | | | | |
| Various timepoints | Derived variable | Height measurements were obtained by trained fieldworkers during nine assessment clinics, from mean age 7.6 to 17.8 years. aPHV was derived from these measurements using Superimposition by Translation and Rotation (SITAR) analysis, described in detail at:  Frysz, M., Howe, L. D., Tobias, J. H., & Paternoster, L. (2018). Using SITAR (SuperImposition by Translation and Rotation) to estimate age at peak height velocity in Avon Longitudinal Study of Parents and Children. *Wellcome open research*, *3*. | | Continuous Measure (in years)  Standardised (z scores) |
| Age at menarche (AAM) | | | | |
| Various timepoints | Puberty 1-9 questionnaires and clinic reports | AAM was obtained using data from postal questionnaires relating to pubertal development, administered approximately annually from age 8 to 17 years. The questionnaires asked whether menstruation had started and if so, at what age (in years and months). Clinical reports were used when questionnaire data was missing. The first-reported AAM was used to minimise recall error. | | Continuous Measure (in years)  Standardised (z scores) |
| Outcome Variables | | | | |
| Body Dissatisfaction | | | | |
| Satisfaction and Dissatisfaction with Body Parts Scale, adapted at age ~14 | ccq220 | How satisfied are you with your____:  Weight | 1 Extremely Satisfied  2 Moderately Satisfied  3 Can’t Decide  4 Moderately Dissatisfied  5 Extremely Dissatisfied  6 Not an Issue  -1 No Response  -10 Not Completed | Continuous Score  Combine ccq222+ccq223 into 1 variable  Replacing missing with mode (2) if at least 9/11 questions NOT missing.  Continuous score generated if at least 9/11 questions NOT missing.  Missing and Not Issue = Missing (6 -1 -10 = .)  Standardised (z scores) |
|  | ccq221 | Figure |  |  |
|  | ccq222 | Build (Males only) |  |  |
|  | ccq223 | Breasts (Females only) |  |  |
|  | ccq224 | Stomach |  |  |
|  | ccq225 | Waist |  |  |
|  | ccq226 | Thigh |  |  |
|  | ccq227 | Buttocks |  |  |
|  | ccq228 | Hips |  |  |
|  | ccq229 | Legs |  |  |
|  | ccq230 | Face |  |  |
|  | ccq231 | Hair |  |  |
| Self Image Profile | | | | |
| Self-Image Profile Scale at age ~14 |  | How often do you feel you are _____: | 1 Always  2 Mostly  3 Sometimes  4 Not Often  5 Never  -1 No response  -10 Not Completed | Binary Variables  Confident, Good Looking (1 2 3 = 1) (4 5 = 0)  Different from others (1 2 = 1) (3 4 5 = 0)  1 Yes  0 No |
|  | ccp107 | Confident |  |  |
|  | ccp111 | Good Looking |  |  |
|  | ccp130 | Different from others |  |  |
| Confounding Variables | | | | |
| Home Ownership | | | | |
| Mother completed; sent when child was 1y9m | g352 | Is your home: |  | Recode (0 2 4 5 = 1) (1 3 6 =0) (-1 7 = .)  0 - House rented from council/housing association/bought from council  1 - House privately rented/ owned/mortgaged |
|  |  | Being bought/mortgaged | 0 |  |
|  |  | Being bought from council | 1 |  |
|  |  | Owned – with no mortgage | 2 |  |
|  |  | Rented from council | 3 |  |
|  |  | Rented from private landlord (furnished) | 4 |  |
|  |  | Rented from private landlord (unfurnished) | 5 |  |
|  |  | Rented from housing association | 6 |  |
|  |  | Other | 7 |  |
|  |  | Missing | -1 |  |
| Maternal Education | | | | |
| Mother completed; sent when child was 5y1m | k6280 | Mother has/is:  No educational quals | 1 Yes  -1 No Response | 0 = < O Level  1 = O Level  2 = > O Level  0 if k6280==1 \| k6281==1 \| k6284==1 \| k6285==1 \|k6286==1 \| k6288==1 \| k6289==1 \| k6290==1  1 if k6282==1  2 if k6283==1 \| k6291==1 \| k6292==1 \| k6287==1 |
|  | k6281 | CSE/GCSE |  |  |
|  | k6284 | Vocational qual |  |  |
|  | k6285 | Apprenticeship |  |  |
|  | k6286 | State enrolled nurse |  |  |
|  | k6288 | City & Guilds Intermediate Technical Quals |  |  |
|  | k6289 | City & Guilds Final Technical Quals |  |  |
|  | k6290 | City & Guilds Full Technical Quals |  |  |
|  | k6282 | O-Level/GCSE |  |  |
|  | k6283 | A Levels |  |  |
|  | k6291 | Teaching Qual |  |  |
|  | k6292 | University Degree |  |  |
|  | k6287 | State Registered Nurse |  |  |
| Major Financial Problems | | | | |
| Mother completed; sent when child was <1, 2 and 5 years old. | f244a | “You had major financial problems” | 1 Yes  2 No  -1 Missing | 0 - No financial Problems  1 - Financial Problems  0 if f244a==2 \| h234a==2 \| k4024==5  1 if f244a==1 \| h234a==1 \| k4024==1 \| k4024==2 \| k4024==3 \| k4024==4 |
|  | h234a |  |  |  |
|  | k4024 | Mother had major financial problems in past year | 1 Yes, affected a lot  2 Yes, moderately  3 Yes, Mildly  4 Yes, did not affect  5 No, did not happen  -1 No Response |  |
| Social Class | | | | |
| Mother completed; sent prior to birth | c755 | Derived Variables  Social Class - Maternal | 1 I  2 II  3 III (Non-Manual)  4 III (Manual)  5 IV  6 V  65 Armed Forces  -1 Missing | 0 if response is 1 2 3 4  1 if response is 5 6  Missing if -1 65  0 Non-Manual  1 Manual |
|  | c765 | Social Class Partner |  |  |
| Sex | | | | |
| Recorded at birth | kz021 | n/a | 1 Male  2 Female  -1 Not known | 1 Male  2 Female |
| BMI at 9 | | | | |
| Various timepoints |  | Calculated based on height and weight measurements obtained from clinics and questionnaire data when clinic data was missing. | | Continuous Measure |
| Auxiliary Variables | | | | |
| Average Weekly Income | | | | |
| Asked when child was 47 months | j410 | “On average, how much is the take home family income each week (include social benefits etc.)?” | 1 < £100  2 £100 - £199  3 £200 - £299  4 £300- £399  5 > £400  -1 Missing | - 1 < £100  2 £100 - £199  3 £200 - > £400  4 £300- £399  5 > £400 |
| Access to a car | | | | |
| Measured at 8 weeks gestation | a053 | “Do you or your partner have the use of a car (including vans, minibuses, etc.)? | 1 Yes  2 No  -7 / -1 Missing | 1 Yes  0 No |
| Crowding Index | | | | |
| Measured at 8 weeks gestation | a551 | Derived: Number of people in the household (a550) divided by number of rooms (a045) | 1 <=0.5  2 >0.5 – 0.75  3 >0.75 -1  4 > 1  -7 / -1 Missing | 1 <=0.5  2 >0.5 – 0.75  3 >0.75 -1  4 > 1 |
| Self Esteem | | | | |
| Measured at 8y6m using 12-item shortened form of Harter’s Self Perception Profile for Children (Harter, 1985) | f8se126 | Derived: Global Self Worth Score: F8 | Global self worth was calculated by summing scores for items 2, 4, 6, 8, 10 and 12. | Continuous measure |
| Stunkard Figures | | | | |
| Measured at 10y8m | cch200 | Child’s perception of his/her body shape | 1 Very thin  2 Thin  3 Average  4 Fat  5 Very Fat  -10 / -1 Missing | Generate score (difference between desired and perceived): abs(cch201 – cch200)  Recode 3,4 = 2 (due to very few obs)  0 No difference (0)  1 Small difference (1)  2 Max difference (2,3,4) |
|  | cch201 | Child’s desired body shape |  |  |
| Carer Completed – Worried about Weight | | | | |
| Measured at 13y10m | ta6160 | “Is he/she upset or distressed about his/her weight or body shape?” | 1 No, not at all  2 Yes, a little  3 Yes, quite a lot  4 Yes, a great deal  9 Don’t know  -11 / -10 / -1 Missing | 0 No  1 Yes  Recode ( 2 3 4 = Yes) |
| Father Absence | | | | |
| Recorded by mother at various timepoints |  | “How old was the child when the natural father stopped living with the child?” |  | 0 Father present  1 Father left after 5 years  2 Father left between 5-10 years |

# S2. Satisfaction and Dissatisfaction with Body Parts Scale, adapted by ALSAPC

| How satisfied are you at the moment with your: | | | | | | |
| --- | --- | --- | --- | --- | --- | --- |
|  | Extremely satisfied | Moderately satisfied | Can’t decide | Moderately dissatisfied | Extremely dissatisfied | Not an issue |
| a) weight | 1 | 2 | 3 | 4 | 5 | 6 |
| b) figure | 1 | 2 | 3 | 4 | 5 | 6 |
| c) body build (breasts in females version) | 1 | 2 | 3 | 4 | 5 | 6 |
| d) stomach | 1 | 2 | 3 | 4 | 5 | 6 |
| e) waist | 1 | 2 | 3 | 4 | 5 | 6 |
| f) thighs | 1 | 2 | 3 | 4 | 5 | 6 |
| g) buttocks | 1 | 2 | 3 | 4 | 5 | 6 |
| h) hips | 1 | 2 | 3 | 4 | 5 | 6 |
| i) legs | 1 | 2 | 3 | 4 | 5 | 6 |
| j) face | 1 | 2 | 3 | 4 | 5 | 6 |
| k) hair | 1 | 2 | 3 | 4 | 5 | 6 |

# S3. Complete Case: Linear Regression Coefficients for the association between pubertal timing (aPHV or AAM) and body dissatisfaction.

|  | **Unadjusted** | | **Adjusted for SES**^A^ | | **Adjusted for SES and BMI** | |
| --- | --- | --- | --- | --- | --- | --- |
|  | Coefficient (95% Cis) | P Value | Coefficient (95% Cis) | P Value | Coefficient (95% Cis) | P Value |
| **Exposure = aPHV (n= 3,184)** | | | | | | |
| Males Body Dissatisfaction | 0.02 (-0.03, 0.06) | 0.523 | 0.02 (-0.03, 0.07) | 0.444 | 0.10 (0.05, 0.15) | <0.001 |
| Females Body Dissatisfaction | -0.11 (-0.16, -0.07) | <0.001 | -0.11 (-0.16, -0.07) | <0.001 | -0.001 (-0.05, 0.04) | 0.937 |
| **Exposure= AAM (n= 2,036)** | | | | | | |
| Body Dissatisfaction | -0.14 (-0.18, -0.09) | <0.001 | -0.13 (-0.18, -0.09) | <0.001 | -0.05 (-0.09, -0.003) | 0.036 |

*Note: One standard deviation increase in aPHV/AAM is equal to the equivalent standard deviation change in body dissatisfaction shown in the table.***^A^** *SES variables include maternal education, social class, home ownership and financial Problems.*

# S4. Complete Case: Logistic Regression Odds Ratios for the association between aPHV and self image profile questions in males and females (N= 3,184).

|  | **Unadjusted** | | **Adjusted for SES**^A^ | | **Adjusted for SES and BMI** | |
| --- | --- | --- | --- | --- | --- | --- |
|  | OR (95% Cis) | P value | OR (95% Cis) | P value | OR (95% Cis) | P value |
| **Males** | | | | | | |
| Confident | 0.97 (0.81, 1.16) | 0.743 | 0.97  (0.81, 1.16) | 0.728 | 0.95  (0.79, 1.15) | 0.616 |
| Good Looking | 1.05 (0.92, 1.19) | 0.483 | 1.04 (0.91, 1.19) | 0.571 | 0.99  (0.87, 1.14) | 0.934 |
| Different from others | 0.88 (0.77, 1.00) | 0.054 | 0.88  (0.77, 1.01) | 0.073 | 0.92  (0.80, 1.06) | 0.232 |
| **Females** | | | | | | |
| Confident | 0.95 (0.82, 1.09) | 0.455 | 0.95  (0.82, 1.09) | 0.440 | 0.90  (0.77, 1.05) | 0.189 |
| Good Looking | 1.11 (1.00, 1.23) | 0.050 | 1.11  (1.00, 1.23) | 0.047 | 1.04  (0.93, 1.16) | 0.521 |
| Different from others | 0.89 (0.79, 1.00) | 0.042 | 0.89  (0.79, 0.99) | 0.039 | 0.92  (0.81, 1.04) | 0.196 |

*Note: One standard deviation increase in aPHV is equal to the equivalent odds ratio change shown in the table.***^A^** *SES variables include maternal education, social class, home ownership and financial Problems.*

# S5. Summary of individual items of the body dissatisfaction scale and Internal Reliability Analysis

|  | Obs | % Dissatisfied | | Item-Test Correlation | Item-Rest Correlation | Alpha |
| --- | --- | --- | --- | --- | --- | --- |
|  |  | **Males** | **Females** |  |  |  |
| Weight | 5875 | 16.1% | 33.2% | 0.806 | 0.748 | 0.900 |
| Figure | 5872 | 15.7% | 28.5% | 0.841 | 0.795 | 0.898 |
| Breasts/Chest | 5838 | 17.4% | 22.3% | 0.549 | 0.455 | 0.915 |
| Stomach | 5855 | 19.2% | 37.4% | 0.85 | 0.745 | 0.901 |
| Waist | 5800 | 14.7% | 25.3% | 0.834 | 0.789 | 0.899 |
| Thighs | 5786 | 13.4% | 44.1% | 0.805 | 0.747 | 0.900 |
| Buttocks | 5712 | 8.8% | 22.7% | 0.752 | 0.692 | 0.904 |
| Hips | 5721 | 8.6% | 22.5% | 0.803 | 0.755 | 0.901 |
| Legs | 5791 | 9.0% | 27.6% | 0.754 | 0.690 | 0.904 |
| Face | 5876 | 10.7% | 22.5% | 0.626 | 0.541 | 0.911 |
| Hair | 5895 | 8.4% | 12.5% | 0.446 | 0.343 | 0.920 |
| Test Scale | | | | | | **0.913** |

# S6. Association between standardised aPHV and individual items of body dissatisfaction scale in Males in complete sample (N=3184).


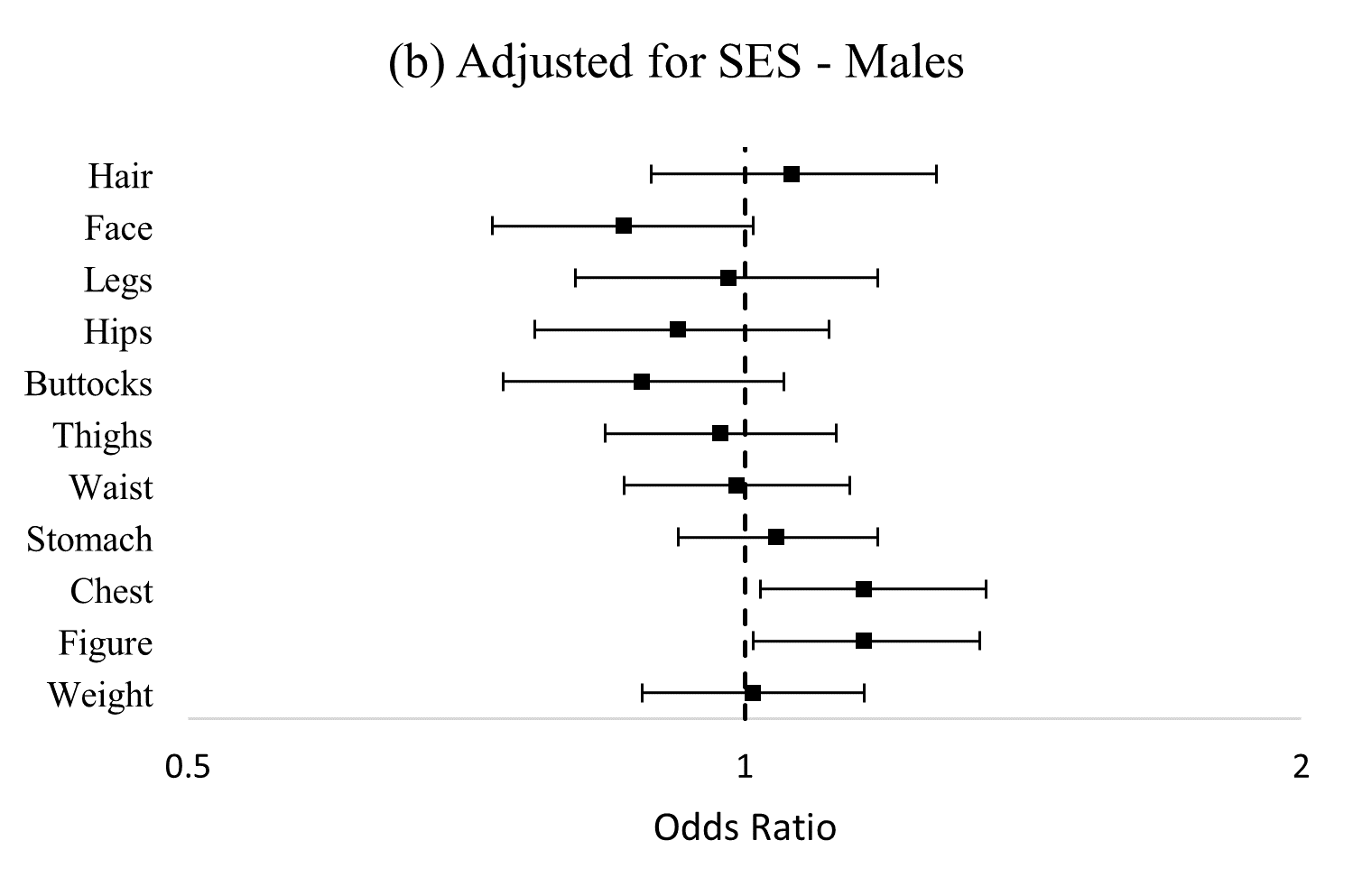


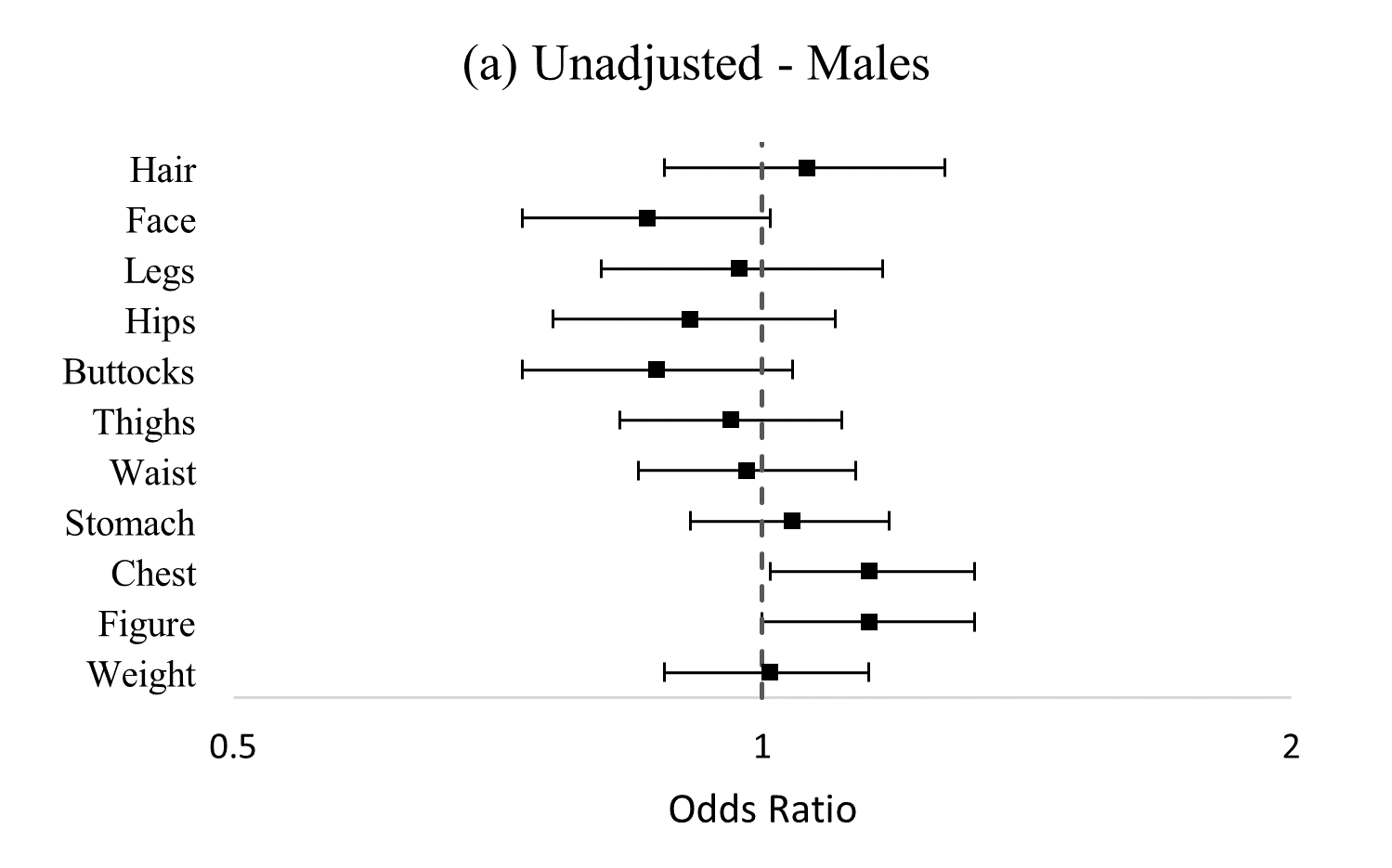


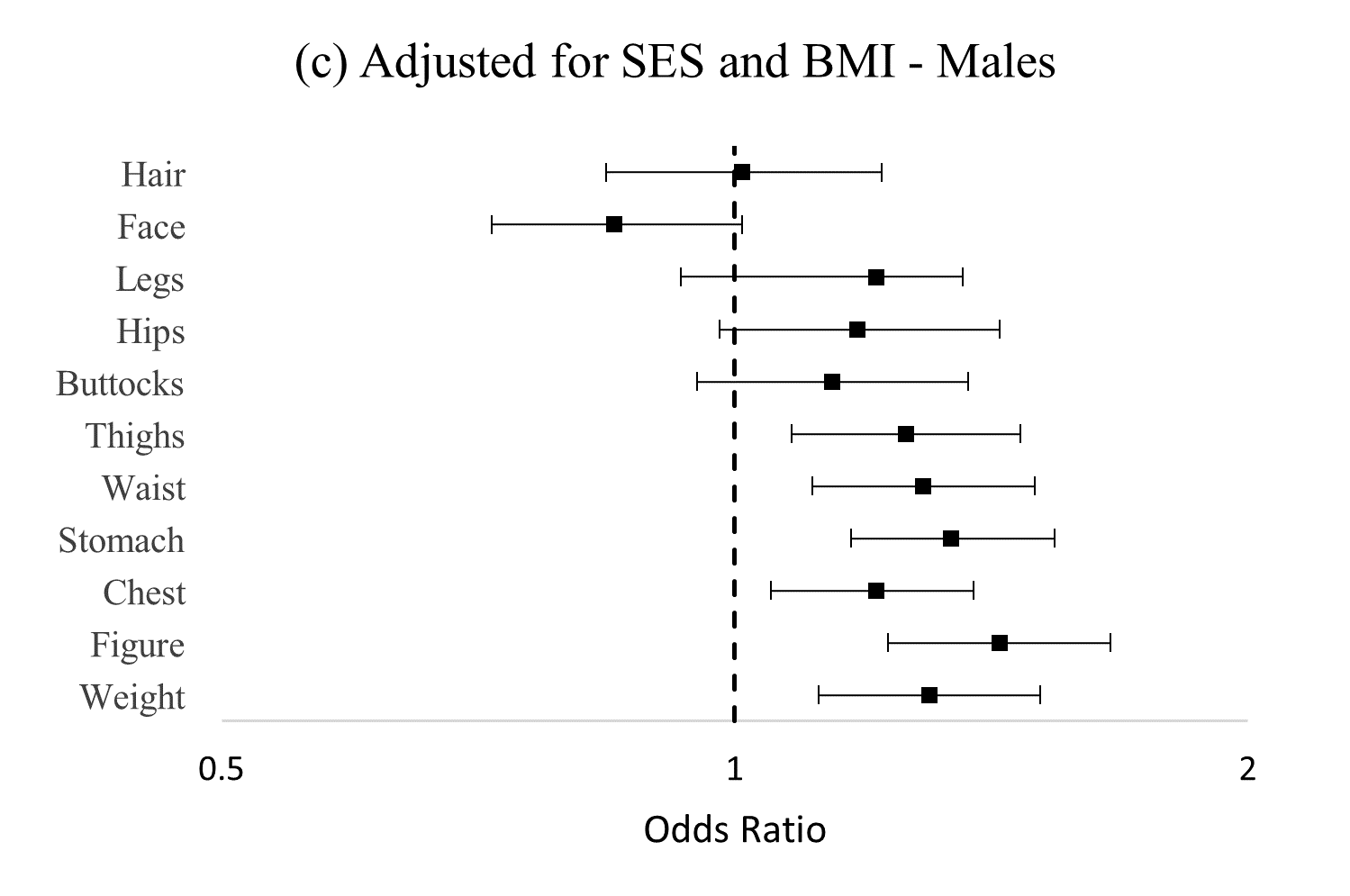


# S7. Association between standardised aPHV and individual items of body dissatisfaction scale in Females in complete sample (N=3184).


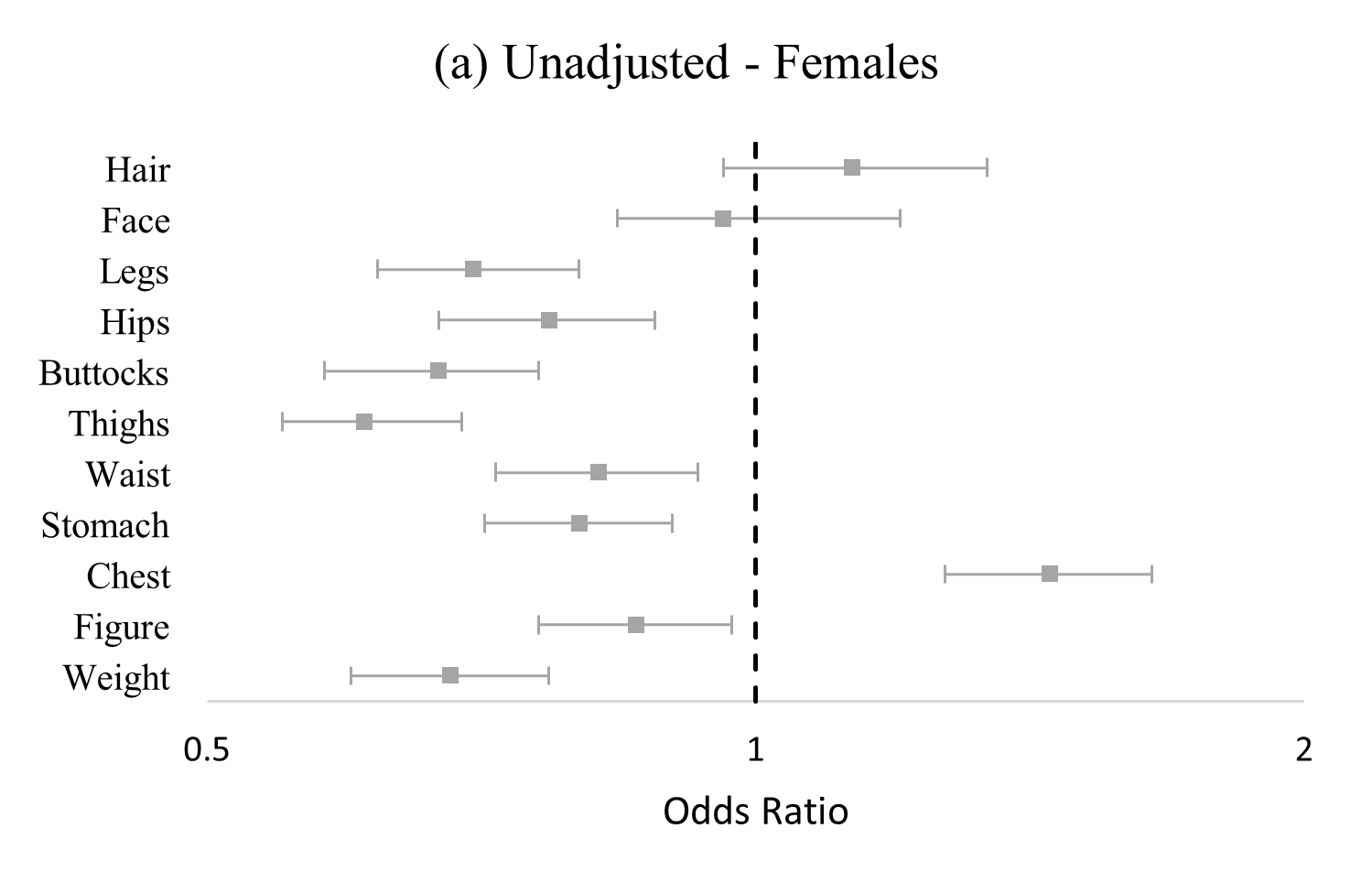


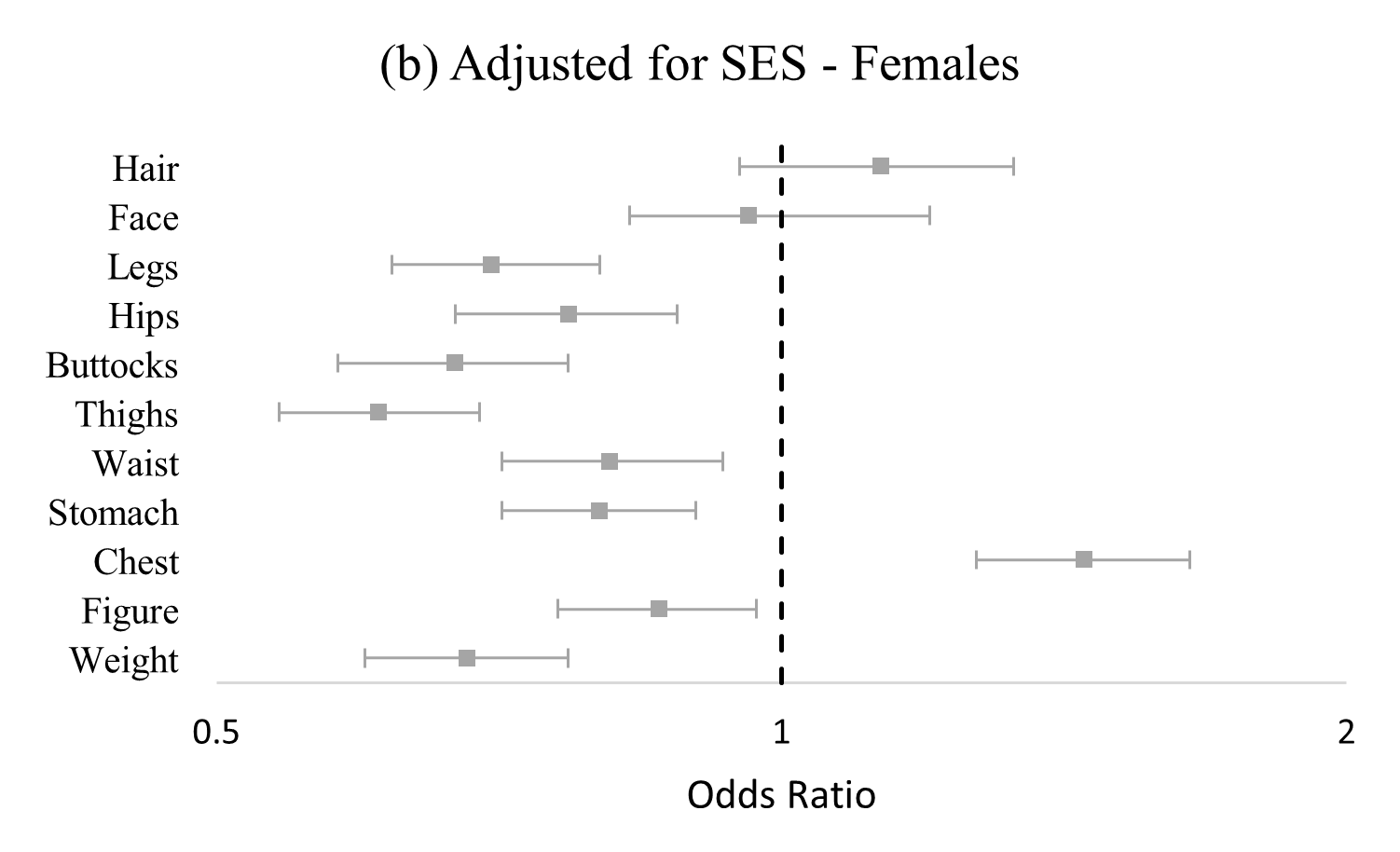


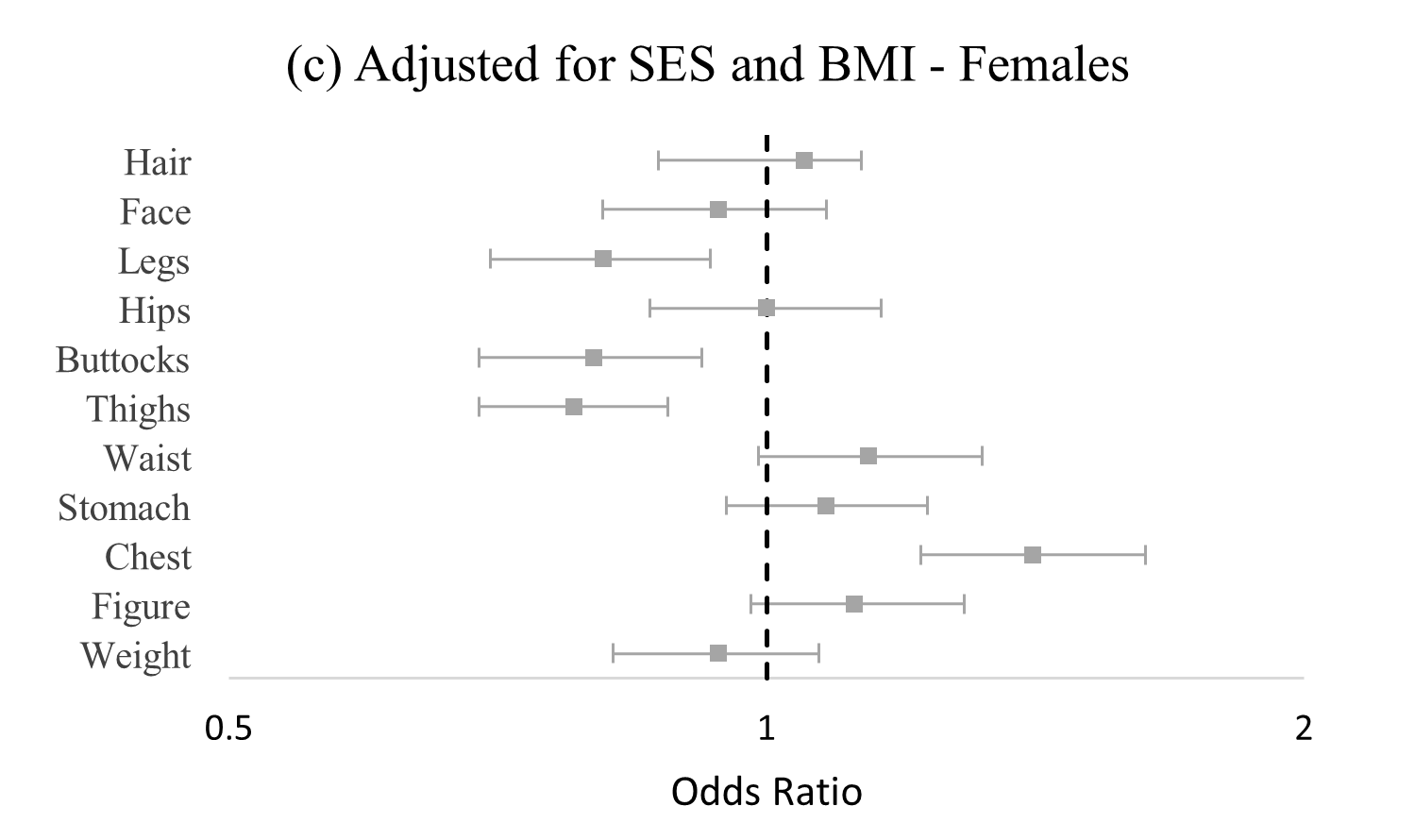

Supplement: online supplemental material 1 [file bmjopen-15-8-s001.docx]
