# Supplementary material for: Pubertal timing, body dissatisfaction and self-image: a prospective cohort study
Source: BMJ Open. 2025 Aug 4;15(8):e092212. doi: 10.1136/bmjopen-2024-092212 (PMC12323538; doi:10.1136/bmjopen-2024-092212)
Supplement: Supplementary file 2 [file bmjopen-2024-092212.reviewer_comments.pdf]

PEER REVIEW HISTORY

BMJ Open publishes all reviews undertaken for accepted manuscripts. Reviewers are asked to complete a checklist review form and are provided with free text boxes to elaborate on their assessment. These free text comments are reproduced below.

ARTICLE DETAILS

Title (Provisional)

Pubertal Timing, Body Dissatisfaction and Self-Image: A Prospective Cohort Study

Authors

Tarif, Dana; Heron, Jon; Fraser, Abigail; Joinson, Carol

VERSION 1 - REVIEW

**Reviewer**	**1**
**Name**	**Bray, Isabelle**
**Affiliation**	**University of the West of England**
**Date**	**02-Sep-2024**
**COI**	**N/A**

Thank you for the opportunity to read this interesting analysis of secondary data which makes a useful addition to the literature. There were some findings which could be touched on in the Discussion:

1. Later puberty in girls was associated with dissatisfaction with breasts and figure - this was not discussed but could be as a result of self-objectification (e.g. https://doi.org/10.1111/j.1460-2466.2012.01667.x)

2. Two measures of pubertal timing are used in the analysis for girls. The findings for age at menarche are stronger, but this is not discussed.

3. Although most associations are attenuated by controlling for confounders (and this is discussed), adjustment for BMI strengthened the association with body dissatisfaction for males only - this warrants discussion.

4. Following on from this, it is interesting that adjustment for SES does not have a notable effect on estimates, but adjustment for BMI does - the discussion could comment on this and consider what other confounders might be important to consider.

In addition, I have a couple of suggestions for the beginning of the paper:

1. In the Abstract, define mid-adolescence as 14 years

2. Keywords - consider removing 'puberty' and adding 'self-image'

**Reviewer**	**2**
**Name**	**Dryer, Rachel**
**Affiliation**	**Australian Catholic University**
**Date**	**29-May-2025**
**COI**	**None.**

Thank you for providing me with the opportunity to review this manuscript. This manuscript was very well written and structured. The data analyses conducted were complex but appropriate given the aims of the study. While this study did not examine a new issue in regard to pubertal timing and body dissatisfaction, it contributes to the existing literature due to the large data set, the control of possible confounding variables, and an examination of individual components of body concerns rather than just an examination of the overall score. I have no recommended revision. Well done.

VERSION 1 - AUTHOR RESPONSE

Reviewer: 1

Comments to the Author:

Thank you for the opportunity to read this interesting analysis of secondary data which makes a useful addition to the literature. There were some findings which could be touched on in the

Discussion:

1. Later puberty in girls was associated with dissatisfaction with breasts and figure - this was not discussed but could be as a result of self-objectification (e.g. https://doi.org/10.1111/j.1460-2466.2012.01667.x)

Thank you for highlighting this point. We have now included a discussion of this finding in the revised manuscript:

> *"Although later pubertal timing in girls was associated with lower overall body dissatisfaction, later puberty was associated with higher dissatisfaction with breasts and figure. This may reflect pressures related to self-objectification, whereby girls evaluate their physical development against societally reinforced ideals of femininity, potentially leading to dissatisfaction (40)."*

2. Two measures of pubertal timing are used in the analysis for girls. The findings for age at menarche are stronger, but this is not discussed.

Thank you for this comment. We have now addressed this point in the Discussion:

> *"In girls, associations between pubertal timing and body dissatisfaction were stronger and more consistent when using AAM compared to aPHV. This may be because AAM is a more salient and socially meaningful marker of pubertal timing, indicating the onset of reproductive capacity and is often seen as a key milestone in female maturation*

(Karapanou & Papadimitriou, 2010). In contrast, aPHV captures a broader growth process which may be less obvious to the individual or peers, and therefore less psychologically salient (Hayward, 2003). As a result, aPHV may have a weaker association with body image concerns, helping explain the observed differences in effect sizes."

3. Although most associations are attenuated by controlling for confounders (and this is discussed), adjustment for BMI strengthened the association with body dissatisfaction for males only - this warrants discussion.

Thank you for this suggestion. We have expanded the discussion to address this point:

> *"Adjusting for BMI attenuated associations between pubertal timing and body dissatisfaction in girls but strengthened this association in boys, suggesting BMI may play a different role in shaping body dissatisfaction between the sexes. In girls, the attenuation supports previous research showing that higher BMI is associated with both earlier pubertal timing and greater body dissatisfaction (Ahmed, Ong, & Dunger, 2009; Paxton, Eisenberg, & Neumark-Sztainer, 2006), indicating that BMI may partly explain this association. In contrast, in boys, adjustment for BMI strengthened the association between pubertal timing and body dissatisfaction. This suggests that BMI may have obscured the strength of the association in unadjusted models. Later maturing boys typically have a lower BMI and may perceive themselves as too lean or underdeveloped compared to peers, which can contrast with the cultural ideal of muscularity (Murnen & Don, 2012; Oehme et al., 2021). This highlights the importance of considering sex-specific body ideals and the complex role of BMI in understanding adolescent body image."*

4. Following on from this, it is interesting that adjustment for SES does not have a notable effect on estimates, but adjustment for BMI does - the discussion could comment on this and consider what other confounders might be important to consider.

Thank you for this suggestion. We have now commented on the differing influence of SES and BMI in the Discussion:

> *"While adjustment for BMI had a notable impact on estimates, adjustment for SES had minimal effect. This may reflect the more direct relationship between BMI and body image concerns, whereas SES may exert a more indirect influence. Although previous research has found that lower SES is associated with earlier pubertal timing {Oelkers, 2020 #90} and greater body dissatisfaction {Paxton, 2006 #91}, these associations are often modest and may not fully capture the psychosocial pathways relevant to body image. Residual confounding by unmeasured factors, such as early life stress and adverse childhood experiences, may also play a role."*

In addition, I have a couple of suggestions for the beginning of the paper:
1. In the Abstract, define mid-adolescence as 14 years
2. Keywords - consider removing 'puberty' and adding 'self-image'

Thank you for your suggestions. We have amended these points to better reflect the focus of the study.

Reviewer: 2

Comments to the Author:
Thank you for providing me with the opportunity to review this manuscript. This manuscript was very well written and structured. The data analyses conducted were complex but appropriate given the aims of the study. While this study did not examine a new issue in regard to pubertal timing and body dissatisfaction, it contributes to the existing literature due to the large data set, the control of possible confounding variables, and an examination of individual components of body concerns rather than just an examination of the overall score. I have no recommended revision. Well done.
